# The Shifting Landscape of Lupus Nephritis Management: A Review

**DOI:** 10.7759/cureus.20950

**Published:** 2022-01-05

**Authors:** Adegbenga A Bankole, Jane N Nwaonu

**Affiliations:** 1 Rheumatology, Virginia Tech Carilion School of Medicine (VTCSOM), Roanoke, USA

**Keywords:** systemic lupus erythematosus, lupus nephritis, lipid disorders, immune, acute tubulointerstitial nephritis, kidney disease, immune mediated side effects, anti-nuclear antibody, auto immune, systemic reviews

## Abstract

Systemic lupus erythematosus (SLE) is commonly the first autoimmune disease that comes to mind for most people when rheumatology is mentioned. It remains an enigma that many of us, including patients and healthcare providers, do not fully understand. Although an ancient disease, it still remains difficult to both diagnose and treat. Historically, there has always been a paucity of therapeutic interventions for SLE as a whole. One of the most distressing manifestations for the patient and diagnostic and therapeutically challenging aspects of SLE is lupus nephritis (LN). There has historically been some difficultly in the development of LN drugs that provide significant therapeutic benefits while having an acceptable side-effect profile. This difficulty led to decades in which no drugs were approved for LN. With a better understanding of the pathogenesis of SLE and LN and improvement in trial design, great therapeutic strides have recently been made. The immunosuppressive landscape of LN has changed recently with the approval of two newer agents as well as a number of promising trials in LN. With the increased number of therapeutic agents (both immunosuppressive and non-immunosuppressive), the clinical question is how and when to use these medications, and, more importantly, which agents to use first. With the increased number of agents, the answers to these questions are becoming more difficult to answer. The purpose of the paper is to review updates in LN diagnosis and management.

## Introduction and background

Systemic lupus erythematosus (SLE) is the archetypical example of an autoimmune disease. It is a clinically and serologically diverse disease that can affect multiple different organs and therefore presents with a variable array of manifestations [1]. Given this, it is sometimes called the great masquerader. Despite the wide range of symptoms, there are well-validated diagnostic tools and criteria used in arriving at a diagnosis of SLE [2]. The incidence and prevalence vary around the world with the highest estimates in North America (23.2/100000 person-years and 241/100 000, respectively) while the lowest incidence is in Africa and Ukraine (0.3/100000 person-years) [3]. Younger women of color are more frequently affected, including African Americans, Hispanics, and Asians, who have the highest incidence and prevalence of SLE in the United States [4]. Patients with SLE suffer significant health risks and have been shown to have nearly double the premature mortality risk of healthy controls [5].

Clinically, SLE can vary from mild disease with musculoskeletal or cutaneous involvement to more severe organ manifestations affecting the renal, central nervous, and cardiovascular systems. Lupus nephritis (LN) is one of the more frequent and most severe manifestations of SLE [6]. The clinical update by Imran et al. noted that around 15%-30% of the patients with lupus were found to have LN at the time of initial diagnosis and 30%-50% developed LN during the course of the disease. This makes LN both a common and severe manifestation of SLE, resulting in significant morbidity and mortality. LN has a cause-specific standard mortality rate (SMR) of 4.689 compared to other complications, including cardiovascular disease SMR of 2.253, and infection SMR of 4.980 [7]. LN also carries a significant risk of end-stage renal disease and all the associated comorbidities [8].

The presentation of LN is variable and may include fatigue, hypertension, edema, proteinuria, abnormal urinary sediment, and abnormal renal function. The clinical picture is divided into nephritic or nephrotic syndrome, though a significant overlap between the two occurs [9]. Laboratory testing, such as urinalysis and microscopy, and immunological parameters can point toward renal involvement and can determine between nephritic and nephrotic disease. A renal biopsy is needed to confirm the diagnosis, to help guide treatment, and even to determine treatment response. LN was first classified histologically by the World Health Organization (WHO) in 1975 [10] and then updated in 1978 [11]. In 2003, the International Society of Nephrology/Renal Pathology Society (ISN/RPS) classification of lupus nephritis was published [12] and this was updated in 2018 [13].

Lupus nephritis is confirmed when there is a positive biopsy in the presence of a positive antinuclear antibody (ANA) test and/or anti-double-stranded DNA (anti-dsDNA) antibody [2]. Some findings on biopsy, including intense C1q staining, full-house staining, extraglomerular deposits, subendothelial and subepithelial deposits, and tubuloreticular inclusions are very specific for LN and are helpful for confirming the diagnosis [14]. LN can be classified into six histologic groups based on the ISN/RPS classification criteria [12]. These classification groupings have clinical relevance and tend to correspond to the two clinical classes (Table 1). The histological groups have a significant impact on therapeutic choices and treatment goals and are of prognostic value. The biopsy findings of the nephritic syndrome are mostly consistent with LN classes ll-lV with those of LN class V being of nephrotic syndrome. As LN classes III and IV carry a 22% risk of end-stage renal disease (ESRD) [8], they tend to be more aggressively managed. The management of LN is divided into the induction and maintenance phases. The goal of induction is to control intrarenal inflammation and immune-mediated activity as rapidly as possible, thereby limiting further parenchymal damage. Maintenance therapy consolidates this and prevents relapse and reactivation of autoimmunity [15].

**Table 1:** Classification of glomerulonephritis in systemic lupus erythematosus

Classification of glomerulonephritis in systemic lupus erythematosus			
	Histology	Presentation	Proteinuria Goal
Class I	Minimal mesangial LN	Asymptomatic/Nephritic	<500 mg/24 hours
Class II	Mesangial proliferative LN	Asymptomatic/Nephritic	<500 mg/24 hours
Class III	Focal LN	Nephritic	<500 mg/24 hours
Class IV	Diffuse LN	Nephritic	<500 mg/24 hours
Class V	Membranous LN	Nephrotic	<1000 mg/24 hours
Class Vl	Advanced sclerosing LN	End-stage renal disease	Not applicable

Given the potential complications, repeating a renal biopsy can be frightening to both the patient and the care provider but can be of great value. A repeat biopsy has been shown to further direct therapy ensuring appropriate treatment while avoiding excessive immunosuppression [16]. Though there is no agreed-upon timing for a second biopsy, the patients should be in complete renal remission for at least 12 months before it is done, as a biopsy at that time will help guide maintenance immunosuppression [17]. It may also be helpful in determining incomplete response to therapy and risk of relapse especially as the clinical and serological activity may not reflect the histological activity seen on the biopsy [17].

## Review

Clinical features

The diversity of the clinical presentations of LN ranges from asymptomatic hematuria/proteinuria, hypertension, overt nephritic and nephrotic syndromes, rapidly progressive glomerulonephritis, and ESRD needing renal replacement therapy. LN, like other more severe manifestations of SLE, is more common in African Americans, Asian/Pacific Islanders, and Hispanic patients [8,18]. LN is a major predictor of morbidity and mortality and may result in the need for renal replacement therapy [8]. Any part of the kidney can be affected by SLE, but the commonest site of renal pathology is the glomerulus [12,19]. The clinical features, treatment, and prognosis vary depending on the site of the pathology. Patients may be asymptomatic, have massive proteinuria, or have rapidly progressive glomerulonephritis leading to ESRD [15].

LN with a nephrotic syndrome commonly presents with proteinuria, peripheral edema, hypoalbuminemia, and dyslipidemia [20]. The levels of hypercholesterolemia and hypoproteinemia/hypoalbuminemia reflect the levels of disease activity in such patients. Studies have shown an association between autoantibodies like anti-dsDNA, anti-histone, and anti-nucleosome antibodies and nephrotic syndrome correspond histologically to ISN/RPS class IV and V [21]. LN with the nephritic syndrome can present with periorbital and pedal edema, hematuria, and non-nephrotic range proteinuria [22] and corresponds most closely to (ISN/RPS class III and IV). As a result of the heterogenicity in the clinical symptoms of LN, the clinical picture may not always correlate with the pathological findings. Other clinical findings can include hypertension, oliguria, and renal insufficiency.

Though not often recognized, dyslipidemia is related to alterations in lipid and lipoprotein metabolism [23]. There is a deficiency and dysfunction of lipoprotein lipase activity, an increased expression of enzymes like acetyl-CoA carboxylase and fatty acid synthase that cause increases in the production of lipids, as well as a downregulation of hepatic lipase activity that results in reduced fatty acid catabolism in the liver [24]. This dyslipidemia also increases the risk of thrombosis in SLE patients who are already predisposed to coagulopathy and can often present as renal vein thrombosis (RVT) and pulmonary embolism [25]. RVT in LN is more commonly associated with membranous lupus nephritis and to a lesser extent with proliferative glomerulonephritis [26]. RVT has also been associated with antiphospholipid syndrome in patients with SLE, and here it can be arterial or venous [27]. As there are no pathognomonic clinical features beyond vague flank pain [28], we should think of RVT if there is an acute worsening in proteinuria, and renal doppler ultrasonography studies can be helpful in arriving at the diagnosis [26], although spiral CT, MRA, and selective venography have a better sensitivity [29]. Although the pathogenesis of renal vein thrombosis in LN remains obscure, nephrotic syndrome is associated with changes in concentrations of factors V, VII, VIII, and X, fibrinogen, and platelets, which result in a hypercoagulable state [30-31].

Autoantibodies

Autoantibody testing is the hallmark of autoimmune diseases, especially SLE and LN. They form part of the diagnostic criteria and can also have prognostic utility. Serum creatinine, glomerular filtration rate, proteinuria, and hematuria are already commonly used as part of the management of LN [32]. Combined with levels of anti-dsDNA antibodies and complements, they are good predictors of long-term renal outcome [33]. However anti-dsDNA antibodies and complements levels are not always abnormal in LN and do not always follow disease activity [34]. A low C4 may reflect a defect in the classical complement pathway and not SLE or LN activity. In fact, the low C4 is also a risk factor for the development of SLE and not just a marker of disease activity [35]. Complement C1q is the first subcomponent of the classical pathway of complement activation and is involved with clearing immune complexes and self-antigens generated during apoptosis [36]. An inherited deficiency in or low levels of C1q is associated with an increased risk of SLE and immune-mediated glomerulonephritis like LN [37]. Autoantibodies to C1q that lower the levels of C1q levels have been shown to closely correlate with LN disease activity levels [38]. Combining the results of the anti-dsDNA and anti-C1q antibody levels enhances the diagnostic specificity and sensitivity for concurrent SLE disease activity [39], and the absence of both anti-dsDNA and anti-C1q has a high negative predictive value for lupus activity.

A number of autoantibodies and serological tests correlate closely with renal pathology and LN disease activity. Anti-dsDNA, anti-nucleosome, anti-ribosome P, anti-C1q antibodies, and C3/C4 follow disease activity. A high titer of anti-C1q or anti-dsDNA antibodies can differentiate LN III and VI from LN V. Anti-C1q has demonstrated a relationship with proteinuria, and this may be higher in LN class V [40].

Antiphospholipid antibodies (aPL) are seen in patients with SLE and LN, and it is not clear that aPL alters the outcomes of LN [41]. However, aPL should be evaluated in all patients with SLE. Some autoantibodies like antibodies to M-type phospholipase A2 receptor (PLA2R) are used to rule out LN. The anti-PLA2R antibody is a specific marker of idiopathic membranous nephritis [42]. There are a number of novel autoantibody tests available that have not been incorporated into routine clinical care.

Treatment

LN is treated with both immunosuppressive and non-immunosuppressive therapies in order to achieve full control. The ACR Task Force Panel on the management of LN recommends that addressing complications/clinical features like hypertension, proteinuria, and dyslipidemia with adjunctive therapy in addition to immunosuppressive therapy leads to the best outcomes [43]. Serum and urine biomarkers are used to monitor therapy, and there is a role for repeated biopsy in the management of LN [32].

Since the discovery of glucocorticoids (GC) in the 1930s-40s, they remain the first drug used in LN. When given at doses above the physiological glucocorticoid levels, they have both anti-inflammatory and immunosuppressive effects [44]. The mechanisms of action of GC are now better understood as genomic mechanisms involving receptor binding, translocation to the nucleus, and binding to DNA binding sites known collectively as glucocorticoid response elements [45], and non-genomic mechanisms of action via the inactivation of the phospholipase A2 enzyme [46]. There are various routes of administration and dosing protocols for glucocorticoids. Induction therapy with high-dose GC is generally accepted as the initial first step in the treatment of LN classes III, IV, and V, with the current, recommended starting doses of 0.5 to 1 mg/kg/d, followed by vaguely defined tapering schedules [47].

Although not a potent immunosuppressive agent, hydroxychloroquine (HCQ) should be used in all SLE patients, including patients with LN. HCQ is protective to the kidneys, reducing the progression of renal damage and SLE and LN flares [48]. As a result of these effects, HCQ remains recommended in the treatment of SLE and LN [49].

Adjunctive therapy

Hypertension should also be addressed aggressively, as it can both be a symptom of LN and cause long-term renal disease. A renin-angiotensin-aldosterone system blockade is recommended in non-pregnant patients, as it has both antiproteinuric and antihypertensive effects [50]. Although all calcium channel blockers (CCBs) are equally effective in lowering blood pressure, non-dihydropyridine CCBs, such as diltiazem and verapamil, have the additional property of reducing proteinuria similar to angiotensin-converting enzyme inhibitors(ACEI) as well as slowing the decline in renal function [51].

Statin therapy should be considered on the basis of lipid levels in the active phase of LN and long-term cardiovascular risk factors. Statins have been shown to reduce proteinuria and thus are a useful adjunct [52]. In addition, statins may have other non-lipid-related immunosuppressive benefits, such as reduction in serum immunoglobulin G (IgG), anti-dsDNA Abs, and proteinuria [53], and can improve long-term outcomes in SLE [54].

Immunosuppressive therapy

Given the varied pathogenesis, presentation, and histology of SLE and LN, it is not surprising that until recently, there has been a lack of drug development resulting in Food and Drug Administration (FDA)-approved medications. After this dearth of new drugs being brought to the market for LN, there have been two medications approved since 2020. Both belimumab (B cell depletory) and voclosporin (a calcineurin inhibitor) are approved by the FDA for the treatment of adults with lupus nephritis.

As early as the 1980s, we understood that LN outcomes were improved with glucocorticoids, however, the dosing varied significantly between different groups. Various studies were performed [55] to determine the ideal dosing. For a number of years, the trend has been to use fewer glucocorticoids, and LN induction therapy is generally accepted as methylprednisolone between 250 mg to 1 g daily for three to five days followed by daily prednisone [56]. The response of LN to glucocorticoid therapy is complicated with multiple side effects and has led to the search for steroid-sparing therapies [57]. Over time, it was understood that a combination of glucocorticoid and immunosuppressive therapy was superior for induction. Intravenous cyclophosphamide (IV-CYC) quickly became the standard of care. Studies confirmed better outcomes in patients on IV-CYC plus glucocorticoids versus glucocorticoids alone [58]. IV-CYC is difficult to handle medically and was not always convenient for patients due to its route of admission, laboratory requirements, and side effects. This lead to the search for other therapies and since 2005, it has been overall accepted that Mycophenolate mofetil (MMF) is the generally preferred agent for both induction and maintenance therapy in proliferative LN.

MMF showed superiority when compared to IV-CYC in its ability to induce remission and in its side-effect profile [59]. When compared with IV-CYC, MMF reduced the risk for renal failure, and this information supported the use of MMF as the first-line induction and maintenance therapeutic agent in LN [60]. MMF remains the cornerstone of LN treatment up until today. Ethnicity and even geographical region seem to have a significant role in the effectiveness of MMF in LN classes III-V, with MMF being more effective in African descent and Hispanic patients with LN when compared to IV-CYC [61]. MMF has been dosed at 2000 mg/day for six months followed by 1000 mg/day or 1000 mg three times daily [59,62]. Initially based on anti-rejection studies, it is currently accepted that patients of African descent benefit from the higher dose of 3000 mg/day [63]. There is still a role for induction therapy with IV-CYC for patients who do not achieve remission or low disease activity on MMF. There is a trend toward lower doses of IV-CYC (500 mg every 2 weeks X 6 doses), with long-term data confirming that these lower doses result in durable remission [64].

For the more difficult patients to treat, tacrolimus at a dose of 0.06-0.1 mg/kg/day can be used. When compared to MMF, tacrolimus is equivalent to the ability to achieve remission [65]. Tacrolimus, a calcineurin inhibition (CI), can be used in combination with MMF to further increase the rates of remission [66]. Given the side-effect profile of tacrolimus, it does need to have drug monitoring and should be kept within a narrow therapeutic index [67]. Voclosporin, another CI that is structurally similar to cyclosporine A has an improved pharmacokinetic profile compared to other CIs. It also does not affect the serum levels of MMF. Through the inhibition of calcineurin, voclosporin blocks IL-2 expression, reversibly inhibits T-lymphocytes and T-cell-mediated immune responses, and stabilizes podocytes in the kidneys. This, in turn, reduces inflammation and renal hemodynamics, treating and improving renal glomerulonephritis associated with SLE. It has shown both safety and superiority when used as part of a combination regimen [68]. Voclosporin can be used in combination with MMF as part of an induction protocol. It shows steroid-sparing properties and has a large effect on reducing proteinuria when dosed at 23·7 mg twice daily [68].

Maintenance therapy has been best studied with MMF and azathioprine (AZA), each having different advantages and disadvantages. MMF is superior to AZA in maintaining remission [69], with neither drug showing superiority in the side-effect profile. In the United States due to the teratogenic side effects, MMF has a black box warning regarding its use in pregnancy. For clinical use, there are now in place shared risk evaluation and mitigation strategies to ensure the safe use of MMF [70]. Patients who achieve remission of LN and wish to conceive or become pregnant should be transitioned to AZA given the safety in pregnancy and fact that flares of LN on AZA remain rare [71]. Belimumab is a recombinant human IgG-1 lambda monoclonal antibody that inhibits B-cell activating factors. It was approved by the FDA initially for the treatment of SLE, and subsequently also approved in December 2020 for the treatment of adults with active lupus nephritis [72]. The addition of Intravenous belimumab at a dose of 10 mg/kilogram of body weight to standard therapy over 104 weeks showed an increase in the number of patients who achieved a primary efficacy renal response [72].

At this time there is no current evidence to guide the choice between belimumab and voclosporin. However, as voclosporin has renovascular benefits, it is a better choice in patients with nephrotic syndrome.

There are other therapeutics in various phases of investigation, including anifrolumab (IFN-α receptor blocker), obinutuzumab (monoclonal anti-CD20), CFZ533X2202 (anti-CD40-CD40L), BMS-986165 (tyrosine kinase 2 inhibitor, blocks IL-12/23, interferon), and KZR-616 (targeted inhibition of immunoproteasome).

**Figure 1:**
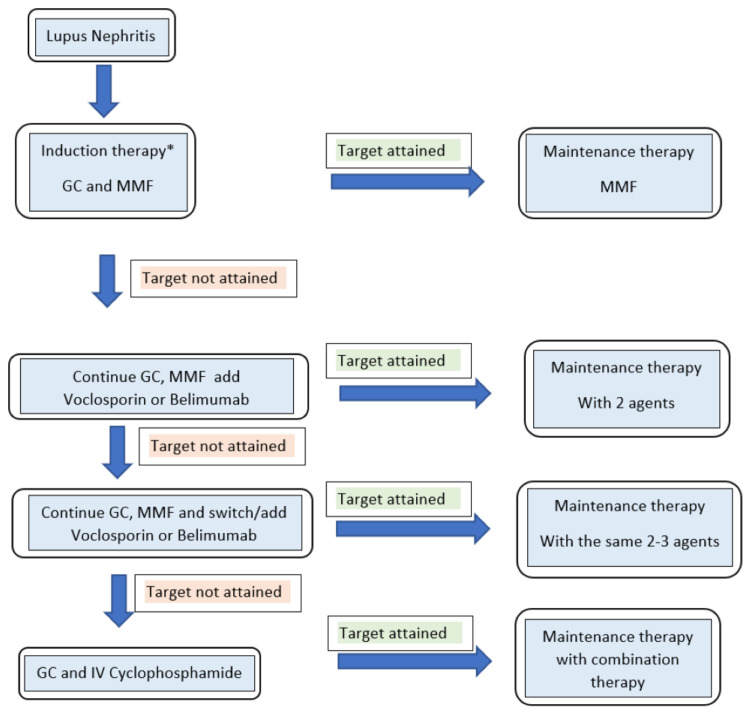
Treatment algorithm for lupus nephritis Treatment algorithm for LN *patient to be initiated on hydroxychloroquine, angiotensin-converting enzyme inhibitors or calcium channel blockers, statins, and adequate blood pressure control Abbreviations: GC, glucocorticoids, MMF, mycophenolate mofeti

Combination therapy is the standard of care in LN and with the increased number of choices, making the right initial decision has become more complicated. The methodical approach that is generally followed in our practice is outlined in Figure 1. At present, it is not the standard of care to use a combination of biological agents, but biological medications in combination with other medicines with different mechanisms of action (MOA) are becoming commonplace [73].

## Conclusions

Our review gives a comprehensive overview of the current understanding of LN and its treatment. It highlights the role of history, physical examination, and laboratory testing, as well as the importance of early diagnosis and treatment, the role of continuous monitoring of response to therapy, and the identification and treatment of complications of LN in reducing poor outcomes. It discusses the more recently approved therapies and outlines a treatment approach that makes use of various medications and their MOA. Despite the recent approval of two new drugs for LN, treatment is still difficult and is complicated by intolerance of medications, access, and, on occasion, compliance, given the large number of medications that may be needed.

As a result, despite the recent improvement in patient outcomes in SLE, renal involvement has a significant effect on the morbidity and mortality of patients, many of whom are women of color in the prime of life. Lupus nephritis is one of the most severe manifestations of SLE that can still result in poorer outcomes, including ESRD and even death.
